# Rare Variants Create Synthetic Genome-Wide Associations

**DOI:** 10.1371/journal.pbio.1000294

**Published:** 2010-01-26

**Authors:** Samuel P. Dickson, Kai Wang, Ian Krantz, Hakon Hakonarson, David B. Goldstein

**Affiliations:** 1 Institute for Genome Sciences and Policy, Center for Human Genome Variation, Duke University, Durham, North Carolina, United States of America; 2 Bioinformatics Research Center, North Carolina State University, Raleigh, North Carolina, United States of America; 3 Center for Applied Genomics, Children's Hospital of Pennsylvania, Philadelphia, Pennsylvania, United States of America; 4 Division of Human Genetics, Children's Hospital of Philadelphia, Philadelphia, Pennsylvania, United States of America; 5 Department of Pediatrics, University of Pennsylvania School of Medicine, Philadelphia, Pennsylvania, United States of America; Medical Research Council Human Genetics Unit, United Kingdom

## Abstract

Genome-wide association studies (GWAS) have now identified at least 2,000 common variants that appear associated with common diseases or related traits (http://www.genome.gov/gwastudies), hundreds of which have been convincingly replicated. It is generally thought that the associated markers reflect the effect of a nearby common (minor allele frequency >0.05) causal site, which is associated with the marker, leading to extensive resequencing efforts to find causal sites. We propose as an alternative explanation that variants much less common than the associated one may create “synthetic associations” by occurring, stochastically, more often in association with one of the alleles at the common site versus the other allele. Although synthetic associations are an obvious theoretical possibility, they have never been systematically explored as a possible explanation for GWAS findings. Here, we use simple computer simulations to show the conditions under which such synthetic associations will arise and how they may be recognized. We show that they are not only possible, but inevitable, and that under simple but reasonable genetic models, they are likely to account for or contribute to many of the recently identified signals reported in genome-wide association studies. We also illustrate the behavior of synthetic associations in real datasets by showing that rare causal mutations responsible for both hearing loss and sickle cell anemia create genome-wide significant synthetic associations, in the latter case extending over a 2.5-Mb interval encompassing scores of “blocks” of associated variants. In conclusion, uncommon or rare genetic variants can easily create synthetic associations that are credited to common variants, and this possibility requires careful consideration in the interpretation and follow up of GWAS signals.

## Abstract 2

A large number of different common variants has been associated with very modest increases of risk for various common diseases. A simulation study shows that rare variants with much greater impacts on disease risk may be responsible for some of these associations.

## Author Summary

It has long been assumed that common genetic variants of modest effect make an important contribution to common human diseases, such as most forms of cardiovascular disease, asthma, and neuropsychiatric disease. Genome-wide scans evaluating the role of common variation have now been completed for all common disease using technology that claims to capture greater than 90% of common variants in major human populations. Surprisingly, the proportion of variation explained by common variation appears to be very modest, and moreover, there are very few examples of the actual variant being identified. At the same time, rare variants have been found with very large effects. Now it is demonstrated in a simulation study that even those signals that have been detected for common variants could, in principle, come from the effect of rare ones. This has important implications for our understanding of the genetic architecture of human disease and in the design of future studies to detect causal genetic variants.

## Introduction

Efforts to fine map the causal variants responsible for genome-wide association studies (GWAS) signals have been largely predicated on the common disease common variant theory, postulating a common variant as the culprit for observed associations. This has led to extensive resequencing efforts that have been largely unsuccessful [1]–[5]. Here, we explore the possibility that part of the reason for this may be that the disease class causing an observed association may consist of multiple low-frequency variants across large regions of the genome—a phenomenon we call *synthetic association*. For convenience, these less common variants will be referred to here as “rare,” but we emphasize that we use this term loosely, only to refer to variants less common than those routinely studied in GWAS.

**Figure 1:**
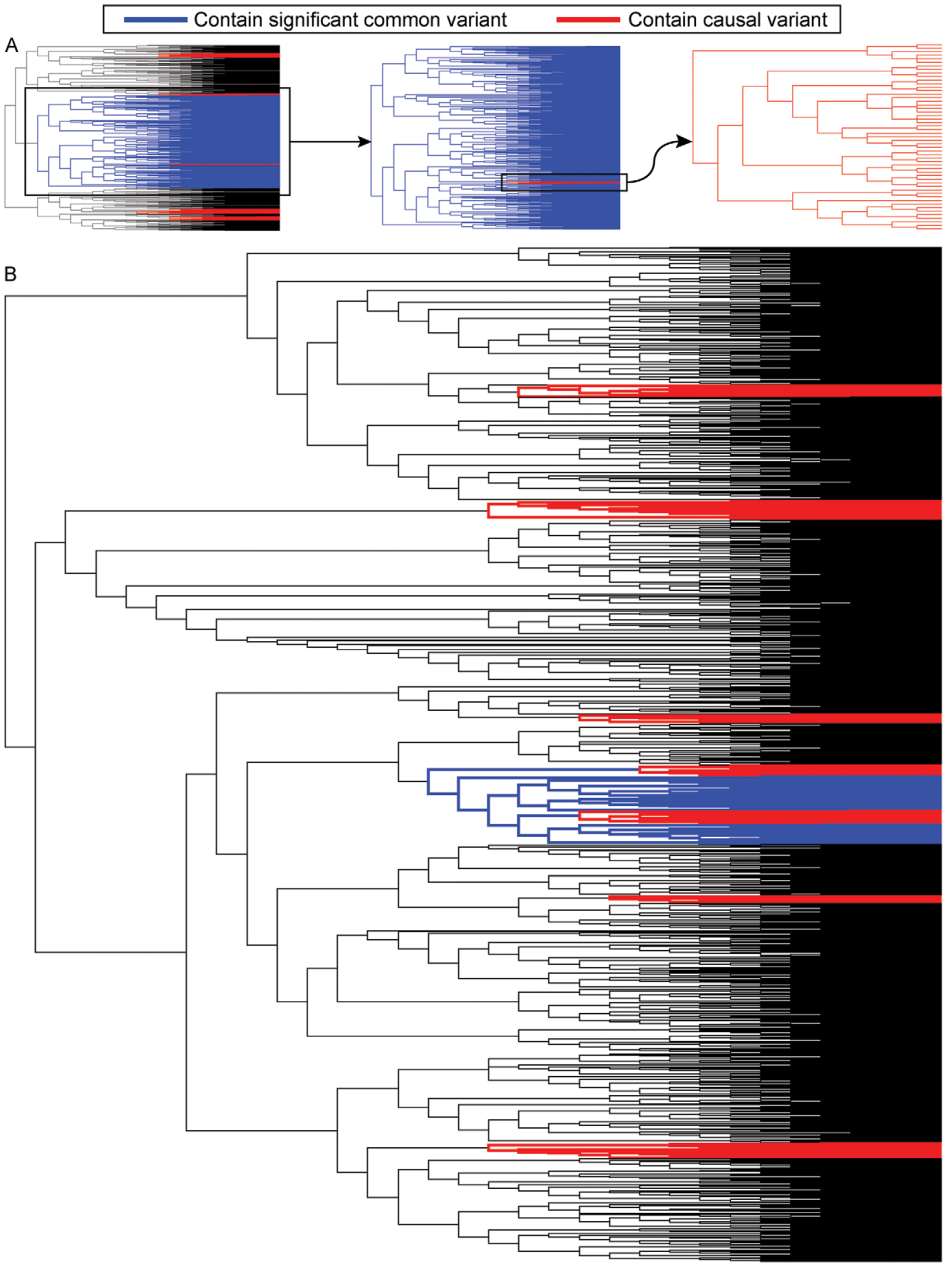
Example genealogies showing causal variants and the strongest association for a common variant. (A) A genealogy with 10,000 original haplotypes was generated with 3,000 cases and 3,000 controls, genotype relative risk (γ) = 4, and nine causal variants. The branches containing the strongest synthetic association are indicated in blue. The branches containing the rare causal variants are in red. (B) A second genealogy was generated using the same parameters. These genealogies demonstrate two scenarios with genome-wide significant synthetic associations: the first (upper genealogy) had a high risk allele frequency (RAF = 0.49), and the second (lower genealogy) had a low RAF (0.08).

The basic idea of how synthetic associations emerge in this model is illustrated in Figure 1, which shows how rare variants, by chance, can occur disproportionately in some parts of a gene genealogy. Any variant “higher up in the genealogy” that partitions those parts of the genealogy containing more disease variants than average will be identified as disease-associated. It is well appreciated that a noncausal variant will show association with a causal variant if the two are in strong linkage disequilibrium (LD). We use the previously introduced term *synthetic association* [6], however, to describe how such indirect association can occur between a common variant and at least one and possibly many rarer causal variants. Using the term *synthetic* as opposed to *indirect* emphasizes that the properties of the association signal are very different when the responsible variant or variants are much less frequent than the marker that carries the signal, as we detail below.

To assess the tendency of rare disease-causing variants to create synthetic signals of association that are credited to single polymorphisms that are much more common in the population than the causal variants, we have simulated 10,000 haplotypes based on a coalescent model in a region either with or without recombination (Materials and Methods). We assumed that gene variants that influence disease have an allele frequency between 0.005 and 0.02, which is generally below the range of reliable detection (either by inclusion or indirect representation) using the genome-wide association platforms currently in use. We assumed a baseline probability of disease of φ for individuals with none of the rare genetic risk factors. The presence of at least one rare risk allele at the locus increased the probability of disease from φ to γ. We considered two values of φ (0.01, 0.1) and chose values of the penetrance γ such that the genotypic relative risk (GRR) of the rare causal variants varied incrementally between 2 and 6, where GRR is the ratio γ/*φ*. These values were chosen to explore the space around a GRR of 4, a threshold above which consistent linkage signals would be expected [7]. We simulated scenarios with one, three, five, seven, and nine rare causal variants.

## Results

**Figure 2:**
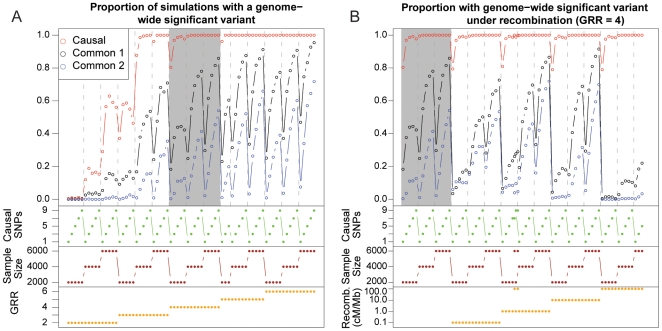
The proportion of simulations with a variant of genome-wide significance. Results for rare variants are shown in red; for the top hit among common variants, results are shown in black; and in blue are the results for the next best hit for common variants after including the top hit in the regression model. At the bottom of each graph, the simulation parameters are represented graphically. Results across all parameters with no recombination are shown in (A) with the shaded region representing the effect size at which linkage analysis is expected to begin generating consistent signals (GRR = 4). Results for simulations that included recombination are shown in (B). The shaded region in (B) is the same as the shaded region in (A), with the rate of recombination for the same parameters increasing along the *x*-axis.

**Figure 3:**
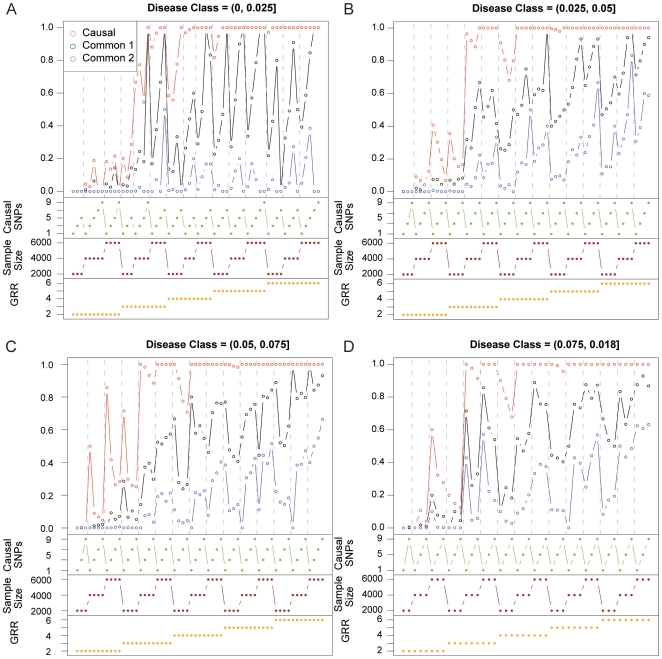
The proportion of simulations with a variant of genome-wide significance separated by disease class. Increasing the number of causal variants generally increases the probability of creating synthetic associations by increasing the size of the disease class without increasing the allele frequency of causal variants. Within disease class, increasing the number of causal variants decreases the probability of creating synthetic association.

Across the conditions we have studied, not only is it possible to achieve genome-wide significance for common variants when one or more rare variants are the only contributors to disease, it is often the likely outcome (Figure 2). Overall, 30% of the simulations were able to detect an association with a common SNP at genome-wide significance (*p*<10^−8^). Three factors—GRR, sample size, and the number of rare causal variants—had a notable impact on power to detect an association with a common SNP. As expected, greater proportions of synthetic associations were created when GRR increased for the rare causal variants and when sample size increased. As the number of rare causal variants increased, the probability of creating a synthetic association did as well. One possible explanation for this increase due to increasing the number of rare causal variants is that adding more causal variants increases the size of the disease class, which is the proportion of haplotypes that carry one or more disease alleles [8]. The size of the disease class varied in the simulations both because the frequency of causal variants was allowed to vary, and because the disease class increases on average with the number of causal variants. To investigate the effect of the disease class on synthetic associations, we separated the results by size of disease class and found first that the larger the disease class the higher the chance of a significant synthetic association. We also find, however, that within a disease class size, the probability of significant synthetic associations decreases with the number of causal variants (Figure 3).

Importantly, association with the strongest causal variant in individual simulations was more significant than with the strongest common synthetic association in 98% of the simulations, and for each combination of parameters, the proportion of simulations with genome-wide significant associations was always higher for the strongest causal variant than for synthetic associations when testing for association with individual variants. Of particular importance to note, except for the case of GRR = 2, all conditions considered here produced a nonnegligible proportion of simulations with significant common variants. It is also noteworthy that significant signals of association can be credited to common variants even when there is only a single rare causal site. A control simulation was run by testing the common variants from one genealogy against phenotypes generated by a separate genealogy with the same parameter settings and not a single test fell below genome-wide significance of 10^−8^ for all simulations. This shows that significant synthetic associations depend on the associations that occur within a single gene genealogy (or correlated ones in a recombination graph) and that sites undergoing free recombination cannot create genome-wide significant synthetic associations.

Intuitively, it seems obvious that when rare variants are the cause of the associations, there should then be multiple common variants that carry significant independent associations. To evaluate this expectation, we took those genealogies that produced a genome-wide significant association and asked what the strongest association was when the top genome-wide significant association was first incorporated in the model. We found that almost 40% of genealogies with a genome-wide significant variant had secondary, independent associations that also achieved genome-wide significance. We also found that fewer than 10% of genealogies had no further significant associations (at α = 0.05). These results demonstrate a clear tendency of rare variants to create multiple independent signals of synthetic association.

One essential question about synthetic associations is whether they are expected to be robust to the presence of recombination. Surprisingly, not only does recombination fail to eliminate synthetic associations, but low rates of recombination can enhance them compared with no recombination (Figure 2B). For example, for GRR = 4 and 9 risk alleles, and a sample size of 3,000 cases and 3,000 controls, we find the proportion of trees showing significance for zero recombination is 0.66. When we introduce a recombination rate of 5×10^−5^ (ten times the genome-wide average for 500 bp) between segments, however, we find that the proportion increases to 0.92. When recombination is increased further, the expected decline in the synthetic association is observed. Importantly, however, even at exceptionally high recombination through the region (5×10^−4^ between segments), we find that almost 30% of the simulations show a significant common variant, and recombination must increase to 5×10^−3^ to reduce the proportion to below 1%. Importantly, the simulations involving recombination prohibit evaluation of any common variant that has a rare causal site within the same segment. Thus the synthetic associations emerging in these simulations occur between sites that are separated by a minimum recombination distance of that between segments, which is 1×10^−3^ to 5×10^−3^. It is counterintuitive that recombination would increase synthetic associations since recombination reduces the average LD in a region. The observation can be explained, however, by the effect of recombination on the distribution of association amongst sites within a genomic region. Although the average LD declines as recombination increases, it is not known how higher moments behave and these moments can influence the proportion of pairs of sites that exceed some given threshold level of association.

**Figure 4:**
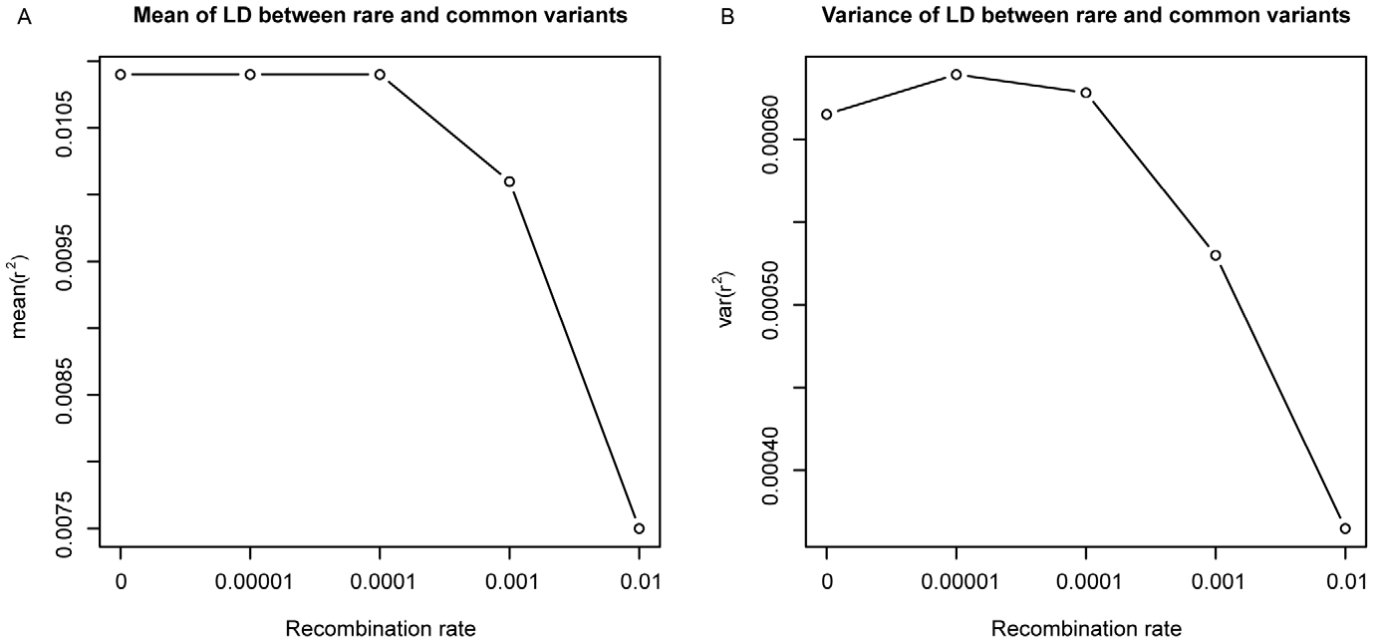
Mean and variance of *r* ^2^ between rare and common sites as a function of rate of recombination. A total of 100,000 simulations of two loci with multiple variants in each loci show how the mean and variance of estimates of *r* ^2^ between rare and common variants are affected by recombination. Although the mean is a nonincreasing function of recombination, the variance increases then decreases, which shows why the maximum *r* ^2^ between rare and common variants can increase with low amounts of recombination in a region.

We tested this as the explanation for the capacity of recombination to enhance associations by directly evaluating the mean and the variance of the association between rare and common variants in a simplified simulation. We considered two regions separated by a specified recombination rate. We calculated the average pairwise association between rare and common variants and also the variance of the pairwise LD between rare and common variants in each simulation, and evaluated both these parameters as a function of recombination. We found that although the mean is nonincreasing, the variance first increases then decreases (Figure 4), suggesting that increases in recombination can “widen” the distribution of LD among sites sufficiently to increase the density in the tail and thereby create stronger synthetic associations.

**Figure 5:**
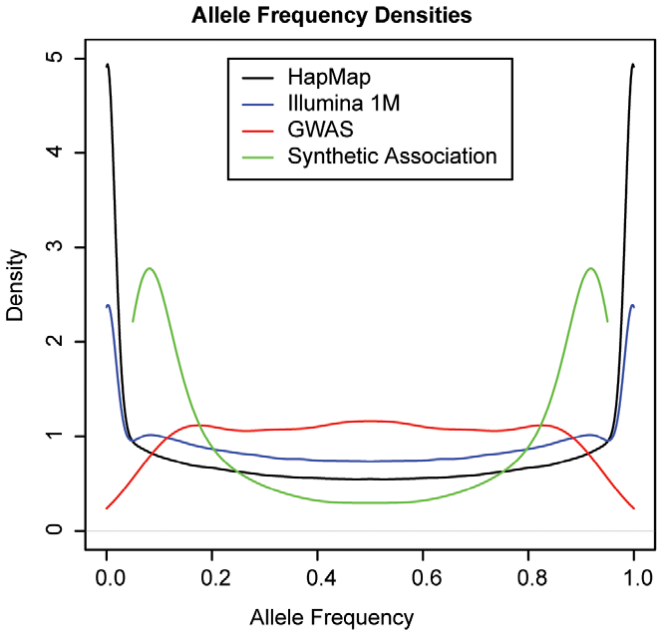
Allele frequency distributions of all HapMap SNPs (black), Illumina 1M SNPs (blue), and GWAS associations in CEU (red), and simulated synthetic associations (green). The allele frequencies show both minor and major allele frequencies. GWAS associations have a clear tendency towards the center, representing greater power to detect association with variants with higher minor allele frequencies. CEU = population of western European ancestry.

These patterns make clear that so long as a given genomic region has one or more rare variants that contribute to disease, these rare variants can generate synthetic associations that are observed in much more common polymorphisms. Under ideal conditions for such synthetic associations, they can be detected with sample sizes far smaller than those routinely used in GWAS. Under less ideal conditions (for example, higher prevalence attributable to environment or to other genetic factors outside of the locus being considered or lower penetrance for the local rare variants), the sample size must be larger. One essential quality of synthetic associations is that although they are often likely to be created when multiple rare variants exist in a region, there are certain conditions under which very little association will be detected even with very large sample sizes and large effects of the causal variants because causal alleles will segregate to opposite common alleles. In other words, no common variant will be able to partition the rare variants on a genealogy to create a large enough imbalance to create association. We also investigated trends in association with causal variants and found that even though our model specified that only derived alleles at causal sites are deleterious, more than a third of the most highly associated common SNPs showed a higher penetrance for the ancestral allele. This result follows observed patterns [9]. Another important trend is that if only rare variants are contributing to the disease class in a region, the risk allele frequency of the most significant synthetic association will tend toward the low end of the distribution of more common allele frequencies (median = 0.10), although over 20% of genome-wide significant synthetic associations had a risk allele frequency above 0.25 (Figure 5). Of course, this trend is noted when all common variants in a region are included, which is not the case with the available commercial genotyping chips, which have a greater probability of including more common variants. In this case, the skew towards lower-frequency variants would be less.

**Figure 6:**
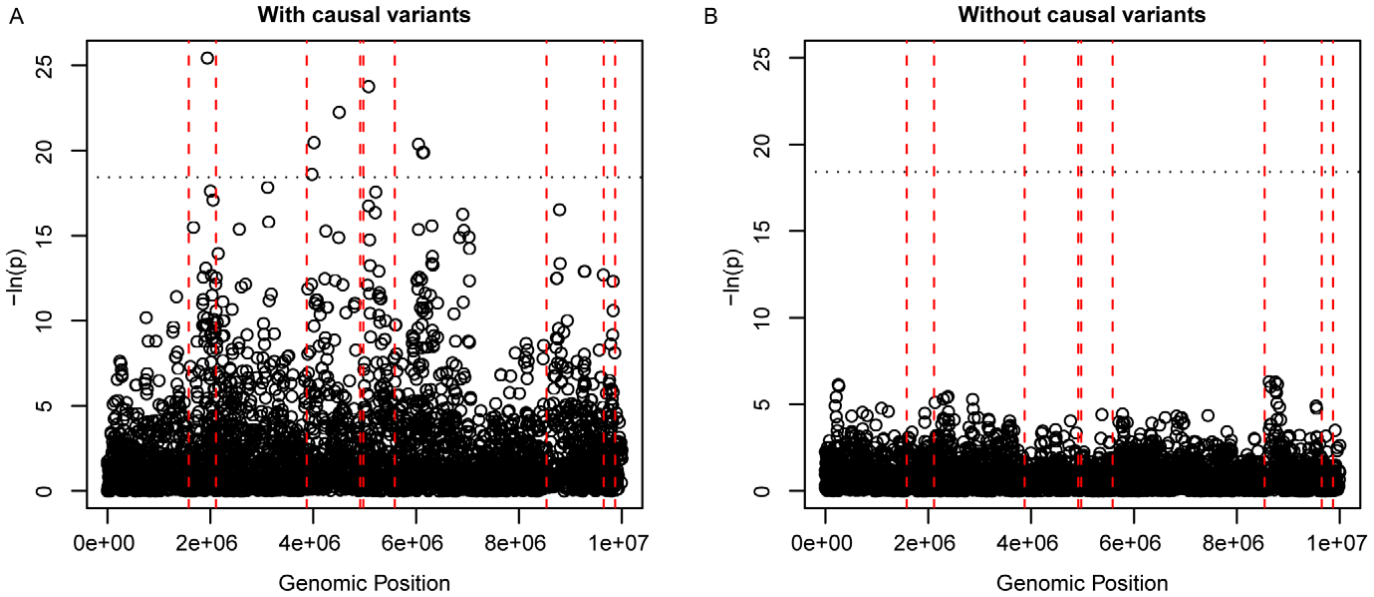
Simulated Manhattan plots in a 10-Mb region. (A) This region has nine rare causal variants selected at random with GRR = 4 and 3,000 cases and 3,000 controls. (B) The same region with permuted phenotypes shows what the region would look like without any association.

We next attempted to determine the expected genomic distances over which rare variants could create synthetic associations. To do so, we simulated a 10-Mb region with a typical recombination rate (1 cM/Mb), nine rare causal variants, 2,000 cases and 2,000 controls, and GRR = 4. We then identified the most distal causal variant that was confirmed to actually contribute to the signal of synthetic association. We did this by finding the most distal variant that resulted in a minimum of a one-log drop in *p*-value when its effect was statistically removed (by incorporation as a covariate into the regression). We found that when a synthetic association reached genome-wide significance, the most distant causal variant that affected the significance of the synthetic association was closer than 2 Mb from a synthetic association in fewer than 13% of the simulations and at least 9 Mb away in 4% of the simulations. The median distance of the most distant causal variant was 5 Mb. A simulated Manhattan plot showing a 10-Mb region with average recombination and nine causal variants with GRR = 4 shows an example of a signature created by synthetic association (Figure 6).

Finally, we evaluated the genomic pattern of synthetic associations using two real-world examples: hearing loss and sickle cell anemia. These two examples represent two possible extremes for synthetic associations. Sickle cell anemia is a serious Mendelian disease in which the body makes sickle-shaped red blood cells. The disease mostly affects subjects with African ancestry, and prevalence among African Americans in the United States is approximately 1 in 600 [10]. It is known to be caused by autosomal recessive mutations in *HBB*, and the frequency of the most common causal variant (Hb S allele) is ∼3.6% in Americans of African ancestry [11]. In comparison, hearing loss is a complex human disease, occurring in one per 1,000 newborns on average [12]. More than two dozen causal genes have been identified for autosomal recessive nonsyndromic hearing loss [13],[14], but mutations in the *GJB2*/*GJB6* locus account for about half of the cases of European ancestry [12],[15]. Among hundreds of known causal mutations in the *GJB2/GJB6* locus [14], the 35delG mutation in *GJB2* is the most common, with an allele frequency of 1.25% in European Americans [16], but hundreds of other point mutations in *GJB2* as well as a 342-kb deletion encompassing *GJB6* also represent known causal variants [17],[18].

**Figure 7:**
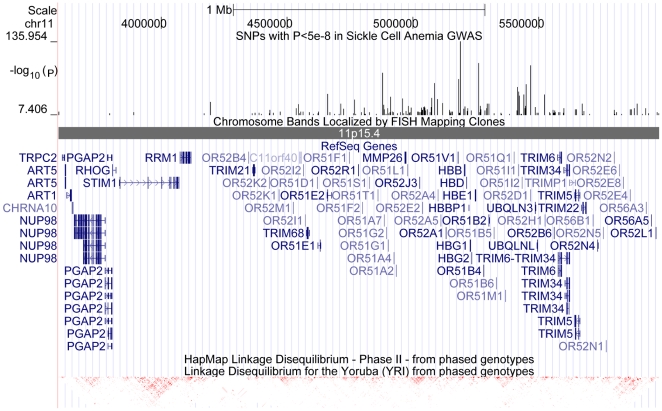
The 2.5-Mb genomic region on chr11p15.4 containing 179 genome-wide significant synthetic associations with sickle cell anemia in African Americans. The −log_10_(*p*) values for all genome-wide significant SNPs were displayed in the upper track, whereas the LD patterns based on HapMap YRI (Yoruba people of Ibadan, Nigeria) population is displayed in the lower track. The region contains dozens of genes spanning several discernible LD blocks.

For sickle cell anemia, a total of 179 SNPs reached genome-wide significance (*p*<5×10^−8^), encompassing an ∼2.5-Mb region on chromosome 11p15.4 (from 3.59 Mb for rs12422109 to 5.98 Mb for rs997433). The region contains dozens of genes and dozens of visually discernable LD blocks in HapMap YRI population. The top association signal (rs7120391, *p* = 1.1×10^−136^) is 9 kb from *OR51V1*, which is very near the causal gene, *HBB* (Figure 7). Clearly, highly significant association signals can travel across multiple LD blocks to distant genomic regions.

**Figure 8:**
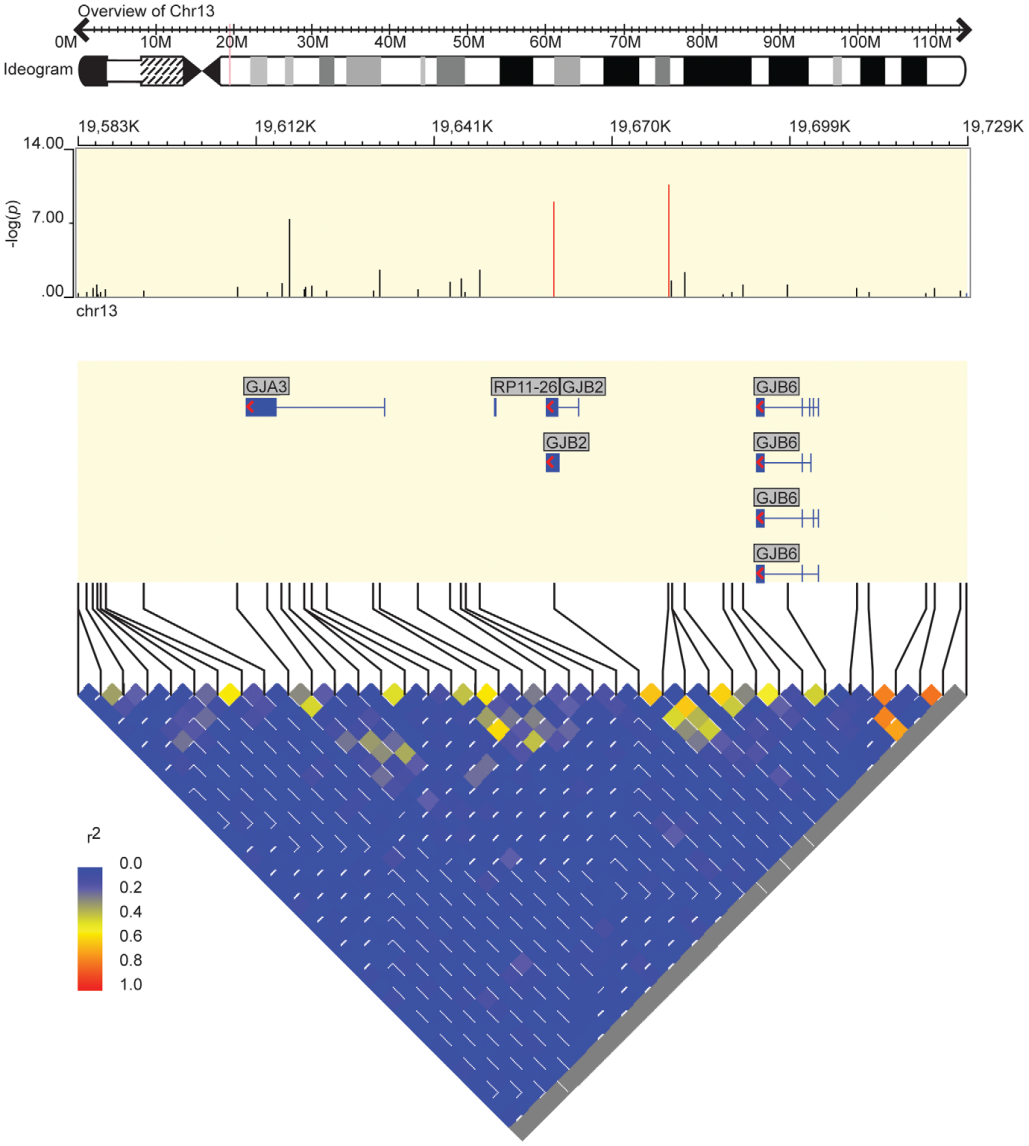
Overview of the GJB2/GJB6 locus on 13q12.11 in the hearing loss GWAS. The three most significantly associated SNPs have weak LD between each other. Although the most common causal variants (35delG) within GJB2 has a frequency of only 1.25% in European Americans, the locus can still be identified by GWAS with common tagging SNPs.

The three most significantly associated SNPs for hearing loss are all located at the *GJB2*/*GJB6* locus on 13q12.1 (Figure 8), including rs870729 near *GJB6* (*p* = 3.38×10^−11^, OR: 1.69), rs3751385 within *GJB2* (*p* = 1.50×10^−9^, OR: 1.63), and rs7329467 within *GJA3* (*p* = 6.87×10^−8^, OR: 1.68). The three SNPs have weak LD with each other (pairwise *r* ^2^ values range from 0.02 to 0.62), but all of them are common variants. For example, rs870729 has a minor allele frequency (MAF) of 18.7% in controls and 28.0% in cases. To evaluate the independence of the association signals from the three SNPs, we tested association again by incorporating rs870729 in a logistic regression model, yet still found residual association for rs7329467 (*p* = 4.3×10^−6^), but not rs3751385 (*p* = 0.33), consistent with the expectations derived above for the behavior of synthetic associations. The locus has been extensively resequenced in numerous studies, and there is no common causal variant at the locus with ∼18.7% allele frequency similar to rs870729. Therefore, rare variants at the locus create multiple independent association signals captured by common tagging SNPs.

## Discussion

These results show that a large proportion of genomic regions that harbor one or more rare variants that contribute to disease is likely to create “synthetic” signals of association [6]. If the region carries an excessively large number of causal variants, this expectation decreases, but for intermediate numbers of (causal) rare variants, detection of many such regions appears inevitable due largely to the fact that increasing the number of rare causal variants increases the size of the disease class in that region.

Separately considering the number of causal variants and the proportion of alleles that are disease causing (the disease class) makes clear that the latter is the key driver of the ease of creating synthetic associations. The intuition for this is obvious. Even when the frequency of disease-causing variants is very low individually, as the disease class grows, collectively they come closer to the frequency of common variants, allowing the possibility of a strong signal to be generated for one of the common variants. This is only prohibited when the causal variants are so numerous as to be distributed roughly evenly through the genealogy (or if an even distribution appears by chance for smaller numbers of causal variants).

In considering the likelihood of rare variants creating a large disease class, it is essential to appreciate that signals can combine in the face of considerable recombination. This makes clear that the “locus” associated with GWAS signals may be far larger than has often been assumed.

We also note that the apparent size of the disease class is not a good guide as to the number of causal variants responsible. Even if the disease class is quite large, it is easily possible that it consists of only rare variants if there are a relatively large number of rare causal variants and these could be spread very broadly over genome regions stretching into the megabases. With respect to the size of the disease class, our simulations highlight the counterintuitive result that, under some genetic models, increases in the number of causal mutations at a locus can increase the probability of a synthetic association.

Although our simulations only show that synthetic associations are likely to occur, coupling this demonstration with the available data does suggest that some of the reported associations are likely to be due to this effect, and many more may be enhanced by the signal of surrounding rare causal variants. First, despite considerable efforts, the vast majority of genome-wide associations have never been tracked to causal sites, even though many surrounding regions have been extensively resequenced [2]–[4]. If all of the responsible variants were common SNPs, one might expect that more clear evidence of causation would have been identified by now for a nontrivial number of common variants. Although this expectation is valid for common causal variants, because we know roughly where to look in the genome, this does not hold for synthetic associations due to rare variants, which may reside at a considerable distance from the associated common variants. Second, it is now known that rare variants contribute to common diseases, and that cases that carry the rare high-penetrant contributors to disease often have “typical” clinical presentations [19]–[21]. On balance, therefore, our results suggest that even though the apparent impact of common variants is only modest for many traits [6],[22],[23], this impact may have been systematically overestimated [24]. It is worth emphasizing that the alternative explanation provided here makes clear, testable predictions. As noted, in a model of synthetic associations, regions that show significant effects for common variants will often consistently show significant residual independent effects after the effect of the most important variant has been accounted for. Second, since rare variants are much more likely to be population specific, synthetic associations are expected to be inconsistent across population groups. In fact, a number of recent studies have confirmed differences in effect between populations [24]–[35]. Table 1 lists variants from these studies in which the point estimate for a follow-up study in a separate population fell outside the 95% confidence interval for the odds ratio of the original study. This includes 13 variants and odds ratios with confidence intervals for the population in which association was first discovered (12 European and one Japanese) and 20 odds ratios for subsequent tests in separate populations, consisting of eight tests in African Americans (seven not significant and one significant in the opposite direction), four tests in Japanese (one not significant and three significant in the same direction), four tests in Koreans (one not significant and three significant in the same direction), two tests in the Indian subcontinent (two not significant), one test in Europeans (not significant), and one in Chinese (not significant). Although it is possible that many of these differences are related to differences in LD (association) between markers and causal sites, genetic or environmental interactions, or simply genetic heterogeneity, it appears likely that many of these differences are due to multiple underlying rare variants that create different synthetic effects in the populations. There are also likely to be other diagnostics of synthetic associations observable in GWAS data. For example, one would expect distinctive extended haplotypes to be enriched in cases relative to controls in large regions surrounding GWAS signals that are synthetic (K. Wang, S. P. Dickson, C. A. Stolle, I. D. Krantz, D. B. Goldstein et al., unpublished data). Perhaps most importantly, the observation that association statistics are stronger for the causal sites in the vast majority of cases implies that in many cases, it should be possible to identify candidate causal sites using whole-genome sequence data surrounding GWAS signals and evaluate these for association. When the association is synthetic, association statistics would be expected to strengthen considerably when the correct causal sites are assayed.

**Table 1:** List of variants in recent GWAS showing evidence of a difference in effect between populations.

						RAF		Sample Size
Trait	SNP	Ethnicity	OR	CI		Control	Case	Control/Case
**T2D**	**rs5015480**	European	1.13	1.08	1.17	0.425	0.379	17,968/14,586
		African American	0.95	0.83	1.08	0.633	0.621	1,054/993
**T2D**	**ra9300039**	European	1.48	1.28	1.71	0.892	0.924	2,432/2,376
		African American	0.42	0.19	0.91	0.889	0.884	1,054/993
		Japanese	1.05	0.94	1.17	0.300	0.350	1,576/1,844
**T2D**	**rs8050136**	European	1.23	1.18	1.32	0.398	0.455	8,284/5,681
		African American	1.02	0.90	1.15	0.446	0.452	1,054/993
		Korean	0.89	0.70	1.14	0.140	0.129	502/908
**T2D**	**rs4402960**	European	1.18	1.08	1.28	0.304	0.341	2,432/2,376
		African American	0.98	0.87	1.11	0.525	0.528	1,054/993
**T2D**	**rs7754840**	European	1.12	1.03	1.22	0.360	0.387	2,432/2,376
		Korean	1.77	1.50	2.10	0.392	0.332	502/908
		Japanese	1.28	1.17	1.41	0.410	0.470	1,576/1,844
**T2D**	**rs17044137**	European	1.16	1.10	1.22	0.230	0.270	2,432/2,376
		African American	0.98	0.86	1.12	0.582	0.615	1,054/993
**T2D**	**rs11037909**	European	1.27	0.97	1.57	0.729	0.760	2,432/2,376
		African American	0.94	0.79	1.13	0.862	0.859	1,054/993
**T2D**	**rs1081161**	European	1.20	1.07	1.36	0.850	0.872	2,432/2,376
		Korean	1.47	1.23	1.75	0.558	0.639	502/908
		Indian Subcontinent	0.78	0.56	1.09	0.912	0.890	516/295
**T2D**	**rs1111875**	European	1.10	1.01	1.19	0.522	0.546	2,432/2,376
		Korean	1.43	1.18	1.72	0.300	0.360	502/908
		Indian Subcontinent	0.93	0.77	1.12	0.465	0.447	514/367
		Japanese	1.27	1.14	1.40	0.280	0.330	1,576/1,844
**T2D**	**rs7923837**	European	1.11	1.02	1.20	0.596	0.622	2,894/2,617
		Japanese	1.27	1.13	1.43	0.190	0.220	1,576/1,844
**Osteoarthritis**	**rs12885713**	Japanese	1.25	1.06	1.49	0.295	0.344	1,006/426
		European	1.01	0.88	1.16	0.582	0.579	752/920
		Chinese	1.00	0.71	1.41	0.205	0.205	210/183
**Breast Cancer**	**rs1219648**	European	1.23	1.03	1.46	0.420	0.470	697/528
		African American	0.84	0.64	1.09	0.450	0.420	427/157
**Breast Cancer**	**rs2981582**	European	1.26	1.04	1.53	0.430	0.470	697/528
		African American	0.80	0.49	1.08	0.520	0.460	427/157

Included are 13 variants and odds ratios with confidence intervals for the population in which association was first discovered, and 20 odds ratios for subsequent tests in separate populations in which the point estimate for the odds ratio in the follow-up study fell outside the confidence interval of the original study.

RAF, risk allele frequency.

There are also practical implications related to finding the variants responsible for observed associations. Perhaps the most important of these is that targeted sequencing within a “block” of LD surrounding GWAS discoveries is often not expected to identify the causal sites. Because modest amounts of recombination can enhance synthetic associations, and because recombination must be exceptionally high to eliminate the possibility of genome-wide significant associations, one or more of the responsible causal sites could be a very considerable distance from the common variant showing a signal of association. This possibility is starkly illustrated by the sickle cell anemia example in which genome-wide significant synthetic associations span ∼2.5 Mb around the causal mutation, although heterosis may also influence this result. This possibility suggests that efforts to identify causal variants responsible for GWAS signals that concentrate on a region of high LD surrounding the implicated variant are not well motivated and are likely to miss many and perhaps most of any rare variants that contribute to synthetic associations (see, for example, [5]). The distance over which synthetic associations occur also offers an alternative explanation to the increasingly common observation of rare variants that occur within the vicinity of a GWAS signal but cannot explain that signal entirely. A simple explanation for such observations is that extending the sequencing to at least 4 Mb and ideally up to 10 Mb around the GWAS signal would pick up other rare variants. In some cases, identifying all the contributing rare variants may explain all of the original signal, whereas in other cases, there could be a combination of rare and common variants contributing. In addition, if synthetic associations are responsible for many of the observed signals, then sequencing in a small number of control samples (even over a much broader genomic region) is also unlikely to succeed. Under our model, the causal sites are both rare and relatively high-penetrant contributors to disease, and will therefore be unlikely to be detected in a small number of control samples. Finally, the focus of attention on genes that are near GWAS signals may be incomplete or misleading in that the actual causal sites may occur in many different genes surrounding the implicated common variant. It is also worth emphasizing that as few as one or two rare variants, at much lower frequency than the associated common SNP, can create a significant synthetic association. In such a case, sequencing a small number of cases that carry the “at risk” common variant might miss entirely the causal rare variants even if the correct genome region is resequenced. These considerations argue for caution in efforts to resequence around genome-wide associations and argue instead that genome-wide sequencing in carefully phenotyped cohorts might be a better use of resources.

It has been suggested that rare high-penetrant variants would produce a signal inconsistent with those observed in many common traits in favor of models with thousands of common variants with marginal penetrance [36]. We have shown that multiple rare variants in a region are capable of acting over large distances to create associations in common variants similar to observed associations. A key point is that multiple rare causal variants may be causing the observed associations, therefore a single haplotype would be insufficient to explain such associations.

Ultimately, the proportion of GWAS signals that is due to common versus rare variants is a question that can only be resolved empirically. Our analyses simply illustrate that in following up GWAS signals, the possibility of synthetic associations must be taken into account. If it were true that many signals were synthetic in nature, however, one interesting and potentially encouraging implication of these results is that some of the very modest associations emerging from genome-wide associations may in fact be pointers to rare variants of much larger effect that could be directly informative about disease pathophysiology or be sufficiently high penetrance to be of useful predictive value.

## Materials and Methods

For the primary simulation, two simulated haplotypes were randomly selected with replacement for each individual, and sufficient individuals were generated to simulate the desired number of cases and controls. Case/control status was designated based on the assigned risk, and equal numbers of cases and controls were selected for association testing. We tested all common variants in the genealogy for association with disease status, where common was defined by a minor allele frequency of 0.05 or greater. Thus we exclude any variant that is actually disease causing and focus on those that are generally represented directly or indirectly in the current genome-wide genotyping platforms [37]. Association tests were performed by comparing 1,000, 2,000, or 3,000 each of cases and controls, and we screened for common variants with *p*-values less than 10^−8^, a now-typical threshold for genome-wide significance [1]. We defined a single “simulation” as follows. A random gene genealogy was drawn with mutations distributed along the genealogy, and disease-causing mutations were assigned at random from those variants that were in the allowed frequency range. Then cases and controls were sampled as described, and the common variants screened for association. We then determined the proportion of such simulations that resulted in a genome-wide significant signal being credited to at least one of the common variants in the genealogy.

Genealogical trees were simulated using GENOME with an effective population size of 10,000 and a mutation rate of 10^−8^ in a 100-kb region. When recombination was simulated, 200 fragments of 500 bp each were used with recombination occurring between each fragment [38]. Trees were drawn using Dendroscope [39].

*p*-Values were obtained using logistic regression on the case-control status under an additive model. Odds ratios (for the common variants) were estimated using the *β* term from the logistic regression. A second *p*-value reported for common variants was based on a logistic regression, with the most strongly associated common variant as a covariate in the model to assess residual association after discounting the strongest synthetic association.

For both disease association studies, we performed a standard GWAS using Illumina HumanHap550 BeadChip with over ∼550,000 SNPs, which represent common tagging variants and do not include any of the disease-causing mutations for either condition. We carried out a standard association test on all markers on the chip passing default quality control measures (minor allele frequency >5%, Hardy-Weinberg equilibrium *p*-value >1×10^−6^, SNP call rate >95%), using the PLINK software [40]. For the sickle cell anemia GWAS, we compared 194 cases and 7,407 controls of inferred African ancestry via multidimensional scaling, with a genomic control inflation factor of 1.01. For hearing loss, we performed a GWAS on 418 cases and 6,892 control subjects, all of whom were of genetically inferred European ancestry via multidimensional scaling, with a genomic control inflation factor of 1.02.

## Abbreviations

GRR: genotypic relative risk
GWAS: genome-wide association studies
LD: linkage disequilibrium
